# Ion Channels and Thermosensitivity: TRP, TREK, or Both?

**DOI:** 10.3390/ijms20102371

**Published:** 2019-05-14

**Authors:** J. Antonio Lamas, Lola Rueda-Ruzafa, Salvador Herrera-Pérez

**Affiliations:** Laboratory of Neuroscience, Biomedical Research Center (CINBIO), University of Vigo, 36310 Vigo, Spain; lolarrzg@gmail.com (L.R.-R.); ssalva4@me.com (S.H.-P.)

**Keywords:** temperature, thermosensors, TREK channels, TRP channels

## Abstract

Controlling body temperature is a matter of life or death for most animals, and in mammals the complex thermoregulatory system is comprised of thermoreceptors, thermosensors, and effectors. The activity of thermoreceptors and thermoeffectors has been studied for many years, yet only recently have we begun to obtain a clear picture of the thermosensors and the molecular mechanisms involved in thermosensory reception. An important step in this direction was the discovery of the thermosensitive transient receptor potential (TRP) cationic channels, some of which are activated by increases in temperature and others by a drop in temperature, potentially converting the cells in which they are expressed into heat and cold receptors. More recently, the TWIK-related potassium (TREK) channels were seen to be strongly activated by increases in temperature. Hence, in this review we want to assess the hypothesis that both these groups of channels can collaborate, possibly along with other channels, to generate the wide range of thermal sensations that the nervous system is capable of handling.

## 1. Introduction

Mammals and other animals spend large amounts of energy in maintaining a nearly constant body temperature, irrespective of the temperature of the environment. The mechanisms controlling thermal regulation are complex and often rely on negative feedback, where it is first necessary to determine the body and ambient temperature. The temperature of the environment can be sensed by external receptor cells, mainly located in the skin, whereas body temperature is sensed by internal receptors expressed by cells located in several internal organs. Traditionally, only the skin and core thermoreceptors (spinal cord, hypothalamus) have attracted the attention of researchers, but more recently, some very interesting information has emerged regarding visceral thermal receptors, even in humans [1,2]. Although a hypothesis conceived many years ago, the terminals of receptor neurons are thought to contain branches of nerve fibers without any apparent structural specialization. Indeed, only recently have we begun to understand the molecular basis of thermoreception by cells.

Many biochemical processes like chemical reactions, and physical processes like conformational changes, are extraordinarily dependent on temperature, and although these processes generally occur faster at higher temperatures, the relationships can be very complex [3]. If we consider the nervous system (NS), the effects of temperature on the resting membrane potential (RMP) were the first to be studied, as were its effects on the kinetics and speed of compound and single action potentials, long before the existence of ion channels was demonstrated [3,4,5,6,7,8].

**Figure 1:**
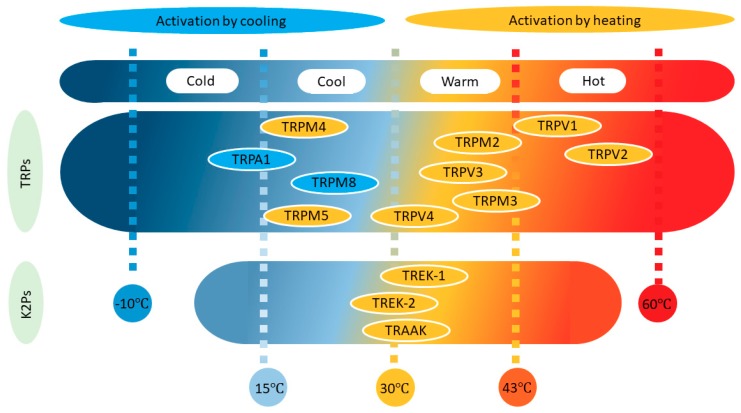
Distribution of transient receptor potential (TRP) and TWIK-related potassium (TREK) channels as a function of their temperature threshold. Note that while TREK channels are activated by increases in temperature (orange), TRP channels may also be activated by lowering the temperature (blue).

All neurons and ion channels are affected by changes in temperature, not least because channel gating is generally a temperature-dependent process [9]. However, only some neurons can be called thermoreceptors and very few ion channel types can be designated as thermosensors. In general, only channels with a temperature coefficient (Q10) ≥2–5 are considered temperature dependent [9,10]. Thermoreceptors are sensitive to changes in temperature rather than to the value of the temperature itself, probably due to their characteristic strong adaptation. These receptors are classified into two groups depending on whether their discharge frequency increases when they are heated or cooled (Figure 1). Based on this classification, it is common to speak of four thermal sensations (cold −10 to 15 °C, cool 16–30 °C, warm 31–42 °C and hot 43–60 °C), whereby cold and hot are potentially noxious and/or painful [11,12].

The modulation of TWIK-related potassium (TREK) channels by temperature has been touched on in several reviews [13,14,15,16,17,18,19,20,21], yet very few have dealt exclusively with this exciting topic [22]. Conversely, after transient receptor potential (TRP) channels sensitive to temperature were discovered, they were studied extensively to understand how thermal stimuli were transduced. Such interest led to the appearance of good reviews covering this issue [12,23,24,25,26,27]. In this review, we will focus on the less well-known role of TREK channels in thermosensation, and we will compare the behavior of these channels to that of TRP channels. Other thermosensitive proteins have also been described, like the Na/K ATPase and ENaC channels, or P2X receptors, and while these should also receive attention, we consider this to fall beyond the scope of this review. Indeed, cell thermosensitivity seems to be governed by the interplay of a number of channel types, as reported in hypothalamic neurons [28].

## 2. TREK Channels

**Figure 2:**
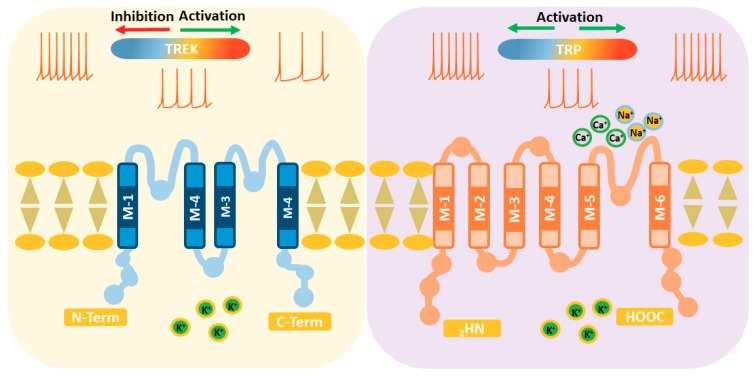
Proteins forming TREK channels have four transmembrane segments and two pore domains so that they assemble as dimers. Activation of TREK channels by increasing the temperature reduces the excitability of thermoreceptors (left). In contrast, TRP channels possess six transmembrane segments and only one pore domain, so that they are tetramers. Activation of TRPs increases the excitability of thermoreceptors, but some are activated by an increase and other by a decrease in temperature (right).

The TWIK-related potassium channel (TREK) subfamily belongs to the two-pore domain potassium channels family (K2P) and is comprised of three members: TREK1, TREK2, and TRAAK (TWIK-related arachidonic acid-activated potassium channel). These are background potassium channels characteristically modulated by several physical and chemical stimuli, such as membrane stretch, pH, unsaturated fatty acids, general anesthetics, and temperature [29,30,31,32,33,34]. In general, TREK channels display very weak activity at room temperature and normal pressure, even when overexpressed in heterologous systems. However, their activity increases strongly when a number of different stimuli are applied, including an increase in temperature [31,35]. From a physiological point of view, it is important to note that at 37 °C, all three members of the TREK subfamily respond to stimuli (pH, membrane stretch, or arachidonic acid), much like they do at room temperature [36]. TREK channels may fulfil a dual role in the transmission of thermal pain. Thus, their strong activation by noxious heat results in an outward current that provokes membrane hyperpolarization and a reduction of thermoreceptor firing, provoking heat-pain relief. Conversely, inhibition of TREK channels by noxious cold should depolarize thermoreceptors and increase their excitability, cooperating in the transduction of noxious cold sensations (see Figure 2).

### 2.1. TREK1

Soon after their discovery, it was shown that TREK1 channels are strongly and reversibly activated by an increase in temperature when expressed in heterologous systems (cell line derived from kidney (COS) cells, oocytes) [31,36]. If we consider that these are mostly voltage-independent channels open at resting potentials, TREK1 channels should function as cold sensors because low temperatures would dampen their activity and depolarize these thermoreceptors (see Figure 2) [31]. Many authors have demonstrated that macroscopic TREK1 currents are strongly outwardly rectifying at room temperature [29,30,32,37,38]. While the outward current is not evident at 12 °C, it is strongly enhanced at 37 °C, and the current progressively increases as the temperature gradually augments [31,39]. Indeed, the current increases around 7-fold with an increase of 10 °C in the range of 14 to 42 degrees, and importantly, maximal sensitivity (0.9-fold per degree) was reached at nearly physiological temperatures, between 32 and 37 °C [31]. The current induced by heating is also outwardly rectifying, and it reverses at potentials close to the equilibrium potential for potassium (E_K_) [31,36,40]. In heterologous systems, the activation of TREK1 by temperature may be reversibly inhibited by cAMP, and this inhibition is suppressed by mutation of the C-terminal region that harbors a phosphorylation site for protein kinase A (PKA) [31]. Moreover, chicken embryonic atrial myocytes express TREK-like currents, and they have a resting membrane potential of around −20 mV in culture, which increases to −70 mV when the temperature rises to 35 °C, a change that was ascribed to the activation of TREK1/2 channels. In voltage-clamp, the outward current recorded at +60 mV increased 9-fold [35]. In fact, both TREK1 and TWIK-related arachidonic acid-activated potassium (TRAAK) channels have been proposed to shut down the firing of hippocampal neurons when the temperature rises too high [28].

The threshold for the activation of slowly conducting C-fibers by noxious heat (30–50 °C) recorded in a skin-nerve preparation decreases in TREK1 KO mice, and the range of activation of these fibers by heat corresponds closely to the range in which TREK1 is activated (30–45 °C) [41]. The number of action potentials in response to a heating ramp (30–50 °C) was higher in the KO mice, although the response of C-fibers to a cooling ramp (32–10 °C) was similar in native and KO mice [41]. Indeed, TREK1 KO mice were hypersensitive to thermal pain up to 50 °C but not at higher temperatures (52–56 °C), indicating that TREK1 channels may be important for the perception of low-threshold but not high-threshold thermal stimuli, for which TRP channels may be more important [41]. Accordingly, the proportion of small diameter, cultured, dorsal root ganglia (DRG) neurons that respond to noxious heat (34%) increases in TREK1 KO (64%) and TREK1/TRAAK KO (74%) mice, as does the proportion of heat-responsive C-fibers in nerve-skin preparations from the single and double KO [42]. It is possible that TREK1 and TRAAK channels counteract the stimulatory effect of heat-activated TRP channels in pain transducing fibers when temperatures increase, such that the overall response may reflect a balance of the activity of these two functionally contrasting channel types. The threshold of thermoreceptors should certainly increase in the presence of TREK channels as temperatures increase.

Cooling of DRG neurons in culture from 32 to 20 °C induces a depolarization of about 10 mV and often the firing of action potentials, an effect shown to be due to the inhibition of a background potassium current [41,43]. Accordingly, the inhibition of a native TREK1-like current may underlie the excitation (depolarization and firing) produced by cold in small, cultured, trigeminal ganglion (TG) neurons (see Figure 2). Interestingly, cold induces subthreshold oscillations in cold-sensitive DRG neurons [44]. Transduction seems to be rather complex, involving the dampening of a hyperpolarization-activated cationic current and a permissive role for a slowly inactivating potassium current [44]. Interestingly, the TREK1/TRAAK double KO mutant shows a consistent cool allodynia, and oxaliplatin, a cancer therapy that causes peripheral nerve neuropathy, exacerbates cold sensitivity in many patients and animals, inducing allodynia to cool temperatures [45].

Neither the deletion of TREK1 or TRAAK increases the fraction of small DRG neurons sensitive to noxious cold stimuli (below 20 °C and down to about 10 °C), although the TREK1/TRAAK KO and the triple TREK1/TREK2/TRAAK KO showed a significant increase in such neurons [42]. Similar results were obtained when recording C-fibers in a skin-nerve preparation, in which case the double KO C-fibers fired more strongly than the single TRAAK KO and wild-type fibers [42]. Oxaliplatin also induced hypersensitivity to noxious cold temperatures [45], while double and triple KO mice but not the TREK1 KO mice are hypersensitive to cold, which is not further affected by oxaliplatin. Hence, the deletion of two of the three TREK channels appears to be sufficient to reach maximal hypersensitization [45,46]. The neuroprotective agent riluzole induces an analgesic effect against painful cold in normal animals, but also in oxaliplatin pretreated TREK2 KO and TRAAK KO animals [46]. However, riluzole did not affect pain sensitivity in TREK1 KO animals treated with oxaliplatin, in animals treated with the TREK1 inhibitor spadin, or in untreated TREK1 KO mice or triple KO animals [46]. Similarly, a presumed TREK leak outward current recorded in DRG neurons was inhibited by riluzole and fluoxetine at 22 and 30 °C but not at 14 °C, probably because the current was already inhibited at low temperatures [47]. Together, these experiments suggest that TREK1 channels fulfil an essential role in the perception of noxious cold and that TREK1 and TRAAK channels work together in sensing cold [46].

Cell-attached patches demonstrated that the basal activity of expressed TREK1 channels is insignificant at room temperature, gradually increasing as the temperature rises (17-fold for an increase of 20 °C) and with a threshold around 25 °C [31,36,48]. The current activated by temperature also displays outward rectification and reverses around the equilibrium potential for K^+^ [31], although the single-channel conductance remains unaffected [36]. TREK1-like channels naturally expressed in cardiac ventricular myocytes and DRGs, and recorded in cell-attached patches, do not open at 24 °C, yet they are very active at 37 °C [36,48]. Surprisingly, increases in temperature fail to modulate TREK1 activity in outside-out and inside-out patches, but under the same conditions, TREK1 is still strongly activated by arachidonic acid [31,36].

TREK1 channels are ideally positioned to act as thermosensors because they are expressed in structures clearly related to thermosensitivity and thermoregulation such as DRGs, the TG, nodose ganglia (NG), or the anterior and preoptic hypothalamus [13,28,31,36,41,47,48,49,50,51,52,53,54,55,56].

### 2.2. TREK2

Heterologously expressed TREK2 channels also produce strong outward rectification when recorded in whole-cell configuration at room temperature [57,58,59], which increases greatly at temperatures around 37 °C in several heterologous systems [36,40,60]. In COS cells, a small TREK2 current was observed at 0 mV that augmented progressively with a gradual rise in temperature to about 40 °C. Notwithstanding, the response of TREK2 to abrupt changes in temperature was rapid [36]. Importantly, the IVs of the TREK2 current at different temperatures (24 and 37 °C) showed that the effect of temperature was not voltage dependent: both inward and outward currents increasing to the same degree. In this range of temperatures, the current increased 14-fold per 10 °C, indicating a very strong temperature dependence that was even bigger than that of TREK1 [36]. Much like TREK1, TREK2 responds to temperature changes around the physiological range, with current activated reasonably well at 37 °C and at resting membrane potential (RMP). Most experiments on TREK channels have been carried out at room temperature and at 0 mV. However, in the future these currents should be investigated using more physiological parameters, around a resting potential and 37 °C, providing a more precise idea of their role in the behavior of central neurons [14].

Cerebellar granule and DRG neurons expressed native TREK2-like channels with weak activity at 24 °C in cell-attached patches, yet when the temperature increased to 37 or 41 °C they became very active at all voltages (−80 to +80 mV) [36,48]. Moreover, cultured cortical astrocytes have TREK2-like whole-cell outward currents that are strongly enhanced in the temperature range of 23–40 °C. Interestingly, ischemia significantly augmented the outward current provoked by an increase in temperature in these astrocytes [61]. In addition, it was recently reported that TREK2 channels contribute about 10 mV to the RMP of DRG neurons at about 30 °C [62]. Furthermore, single TREK2 and triple TREK1/TREK2/TRAAK KO mice were more sensitive to warm temperatures (40–42 °C) when tested with the tail-flick reflex [63].

Using a skin-nerve preparation, it was demonstrated that the proportion of heat-sensitive C-fibers and their activity (number of action potentials) increased in the TREK2 and triple KO mice when temperatures rose to noxious heat levels (ramped from 30 to 50 °C), whereas the temperature threshold for firing decreased [63]. At high temperatures (between 40 and 50 °C), the triple but not the single KO fibers were more active than their wild-type counterparts, indicating that TREK2 regulates C-fiber responses at temperatures below 40 °C, while at higher temperatures other TREK channels participate in these responses [63]. Both KOs suffered hyperalgesia at temperatures around 45 °C, but only the triple KO showed the same behavior above this temperature [63].

The withdrawal latency in the tail immersion test was clearly reduced in both the TREK2 KO and the triple KO mice when innocuous cooling temperatures were tested (20–25 °C). As such, the KOs show enhanced sensitivity to temperatures in the normal range and similar results were obtained in a temperature preference test [63]. The percentage of C-fibers responding to moderate cold (30–21 °C) was clearly higher in single and triple KOs when compared to those recorded from the nerve-skin preparation of wild-type mice [63]. Interestingly, the cold threshold for C-fiber firing (21 °C) was lower in the triple KO (24 °C) but not the TREK2 KO (23 °C) mice [63]. Moreover, oxaliplatin induces mice to spend more time on a hot plate (30 °C) than on a cold plate (20–25 °C) when compared to untreated animals, indicating that neuropathic mice have enhanced sensitivity to moderate cold [63]. It has been suggested that TREK2 is implicated in the neuropathic hypersensitivity induced by this drug and indeed, oxaliplatin almost halved the TREK2 mRNA detected in DRG neurons [63]. Generally, the data suggest that TREK2 channels may be essential for the control of the C-fiber response to cold at moderate temperatures. The tail immersion test showed that triple KO mice were hypersensitive to noxious cold temperatures (15–5 °C), while the single TREK2 KO mice behaved much like the wild-type mice. Moreover, very similar results were obtained in the nocifensive dynamic cold plate test [63]. Accordingly, it was suggested that TREK2 may not be important in noxious cold sensitivity but that it might be essential for thermoreception at moderate cool temperatures (25–20 °C) [63].

A clear, fast, and reversible increase in activity was also reported for single TREK2 channels in cell-attached patches held at −40 mV when the temperature increased (24 to 37 °C), with a threshold for this increase at 25 °C (from 24 °C) and not affecting the conductance [36,48]. It should be noted that in these circumstances, the activity of TREK2 single channels was very low at 24 °C [36]. Significantly, neither TASK3 nor TRESK2 showed such dependence on temperature [36]. However, like TREK1, the activity of TREK2 in inside-out patches was not modified by changes in temperature (24 to 42 °C) [36]. Finally, it is important to consider that TREK2 channels are expressed strongly in the DRG, TG, and hypothalamus [36,47,48,51,52,55,56], yet less than TREK1 in the NG [49].

### 2.3. TRAAK

Much like the other members of the family, TRAAK currents showed a strong open-channel outward rectification when recorded in whole-cell configuration [64,65,66], and these currents increase strongly when the temperature rises (24 to 42 °C) [36]. Moreover, the percentage of small-diameter DRG neurons responding to noxious heat, in culture, is increased in TRAAK and TRAAK/TREK1 KO mice. Consistently in skin-nerve preparations, the percentage of fibers responding to heating (30–50 °C) and the number of action potentials in response to a heating ramp also clearly increases, while the firing threshold is reduced [42]. Notably, TRAAK and TRAAK/TREK1 KO mice suffer heat hyperalgesia when evaluated in the tail immersion test in the 46–50 °C range. Moreover, the double but not the single KO also shows hypersensitivity at higher temperatures (52–56 °C) in the hot plate test [42].

Knock-out of TRAAK did not modify the percentage of DRG neurons in culture that respond to noxious (12 °C) cold [42]. Moreover, in the cold plate assay, TRAAK KO mice behave like wild-type mice, whereas TREK1/TRAAK KO mice are more sensitive to cooling in the 10 to 20 °C range [42]. The activity of single TRAAK channels heterologously expressed in COS cells and recorded in cell-attached patches at −40 mV was very low at 24 °C, yet it increased progressively as the temperature rose from 24 to 37 °C. The threshold for activity was around 30 °C, slightly higher than that reported for TREK1 and TREK2 [36,48]. However, the behavior of TRAAK channels in inside-out patches mimics that of TREK1 and TREK2 such that their activity was not affected by changes in temperature (from 24 to 42 °C) [36]. Native TRAAK-like channels in DRG neurons displayed little activity at room temperature, but there was clear activity in all cell-attached patches at 37 °C [36, 48]. Finally, TRAAK channels are clearly expressed in the hypothalamus, TG, and DRG [28,36,47,48,51,52,55,56,66,67], yet they are only weakly expressed in the NG [13,49].

### 2.4. Molecular Origin of Thermosensitivity

When first discovered, mouse TREK1 was reported to have four transmembrane segments, two pore domains and a sequence of 370 aa [37]. The activation of heterologously expressed TREK1 currents by increasing temperature is unaffected by deletion of the cytoplasmic N-terminal region. By contrast, partial deletion of the C-terminal region (Δ103) or replacement of this region with that of TASK1 strongly dampens the activation of TREK1 by heat [31]. Sensitivity of the TREK1 channels to temperature can be eliminated by mutating helix 1 of the pore (G137I), suggesting that temperature affects the TREK1 and TREK2 channels by manipulating the C-type gate [22]. It was suggested that functional coupling between the C-terminal domain and the C-type gate through the M4 segment is crucial for the heat sensitivity of the TREK1 channel [22]. Thus, it is tempting to speculate that increasing the affinity of the C-terminal domain for phospholipids of the inner leaflet would increase the activity of TREK1 by heat, as proposed for other stimuli like stretch, PUFAs, phospholipids, or pH. Conversely, dissociation of this domain from the membrane would result in TREK1 inhibition [13,21,68,69]. Surprisingly, replacement of the C-terminus of TREK2 with that of TASK3 did not reduce the sensitivity of the channel to changes in temperature in the range of 24 to 37 °C under similar conditions, although it became insensitive to pH and arachidonic acid [36]. Heat enhances the activity of TREK1, TREK2, and TRAAK in whole-cell and cell-attached recordings, yet not in outside- and inside-out patches, indicating that the integrity of the cell, and probably also a second messenger, are necessary for this modulation [31,36]. The contribution of TREK channels to maintaining the RMP has often been questioned; however, this assertion is mostly based on experiments carried out at room temperature. Thus, new experiments should be performed at physiological temperatures to ascertain the role of these channels on both the RMP and neuronal excitability.

## 3. TRP Channels

Six transient receptor potential (TRP) channels are considered thermosensors, four of them responding to heat and two to cool [11,26]. Temperature-sensitive TRP channels (Thermo-TRP) are extremely dependent on temperature, showing very high Q10 values (>20) [70].

### 3.1. Heat-Sensitive TRP Channels

Four TRP subtypes are activated by an increase in temperature (Figure 1). Two of them respond to warm stimuli (TRPV4 Warm >27 °C and TRPV3 Warm >34 °C), and the other two to hot-painful stimuli (TRPV1 Hot >43 °C and TRPV2 Hot >52 °C) [23,25].

TRPV1s are voltage- and temperature-dependent channels that display outward rectification when expressed in human embryonic kidney (HEK) cells and that are strongly enhanced by heating (to 48 °C) and by capsaicin [71,72]. At room temperature, the current passing through these channels is negligible below 0 mV, but at 42 °C the channel activates more or less between −100 and +50 mV [71]. These cationic channels are ten times more permeable to Ca^2+^ than to Na^+^ (P_Ca_/P_Na_ ~10) and are thought to be sensors for noxious heat but not activated by innocuous heat [24,72,73,74]. Indeed, the response to noxious heat in mice lacking TRPV1 (KO) or DRG neurons was clearly weaker, although other channels may also contribute to the perception of noxious thermal stimuli because heat still evokes receptor activation in several preparations [23,25,73]. The NG sensory neurons that innervate the lungs produce an inward current in response to an elevation in temperature (from 23 to 41 °C, with a threshold around 35 °C and a Q10 of about 30 in the range of 35–41 °C) as well as membrane depolarization and action potential firing. This response was ascribed to the presence of TRPV1 channels, even though the participation of TRPV2-4 could not be ruled out [10]. We obtained similar results with NG neurons in culture, although these were slightly more complex because a hyperpolarization was observed before the depolarization and firing (unpublished data). It is interesting to note that inflammatory mediators like ATP and bradykinin strongly reduce the threshold of TRPV1 activation (30 °C) such that warm temperatures become painful [23]. TRPV1 is strongly expressed in small-diameter sensory neurons of the DRG, TG, and NG, but also in the hypothalamus [10,23,72], sites where they may exert an important role in thermoreception.

TRPV2 is activated at extremely high temperatures (52 °C), although it is not affected by capsaicin and shows an outwardly rectifying IV curve and a P_Ca_/P_Na_ ~3 [24,70,75,76]. This channel has a Q10 of around 100, and it is thought that the temperatures that activate TRPV2 are more harmful than those that activate TRPV1 [77]. These channels are strongly expressed by myelinated medium-large diameter DRG neurons (Aδ and Aβ), as well as in the hypothalamus and in the NG [10,78].

TRPV3 channels are activated at warm, close to hot, temperatures (around 34–39 °C, with a Q10 around 6), generating currents with pronounced outward rectification and a P_Ca_/P_Na_ ~12 [24,79]. They are capsaicin-insensitive channels but stimulated by camphor [79], and they are thought to be involved in thermosensation and thermal nociception [79,80]. Indeed, it has been suggested that TRPV3 channels contribute more to the speed with which mice select a more comfortable temperature than to the choice of the value of the temperature itself. By contrast, TRPV4 channels are more likely to be involved in choosing the preferred temperature from a non-painful range [26]. Interestingly, it was proposed that TRPV3 channels transmit thermal stimuli through skin keratinocytes, which in turn will transmit this information to sensory endings [25,81]. TRPV3 channels are expressed in sensory DRG and NG neurons but also in the hypothalamus. Interestingly, they co-localize with TRPV1 in DRG neurons [10,82].

TRPV4 are cationic (P_Ca_/P_Na_ ~6 [24,76]) channels activated at even lower warm temperatures (around 27 °C, with a Q10 of about 10), generating outwardly rectifying currents and responding dynamically to temperature changes in the physiological range [82]. These channels were proposed to play a role in thermosensation and thermoregulation [82,83], although some authors were unable to activate these channels by increasing the temperature. Similarly, some behavioral studies reported a reduced response to temperature changes in TRPV4 KO mice, a behavior that was less clear in other studies [84,85]. Much like TRPV3, the expression of these channels in keratinocytes was proposed to play an important role in the transmission of thermal information, which probably contributed to the controversy generated [25,26,83]. The sensitivity of this channel to temperature is lost in excised patches, suggesting that it requires a soluble intracellular factor [86]. TRPV4 channels are expressed in DRG, TG, NG, and preoptic/anterior hypothalamic [10,82] neurons, although in the hypothalamus they seem to be expressed in terminals rather than in the soma, such that their role in body thermoregulation is unclear [28].

TRPM2 (>35 °C), TRPM3 (>40 °C), TRPM4 (>15 °C), and TRPM5 (>15 °C) are channels that can also be activated by warming (Figure 1), yet they have received less attention, probably because it was initially thought that they were not expressed by somatosensory neurons or keratinocytes [12,26,87]. TRPM2 is voltage-insensitive, shows a P_Ca_/P_Na_ ~1 [24,76], activates at 35 °C, and has a Q10 of around 15 [12,88]. TRPM3 is expressed broadly, generating an outwardly rectifying current, having a P_Ca_/P_Na_ between 0.1 and 10 [24,76], and activating at >40 °C with a Q10 of 7 [89]. It is important to say that TRPM3 has been described as part of a triad of TRPs, together with TRPV1 and TRPA1, involved in the transduction of acute noxious heat in mice [90]. The combined ablation of these channels (triple KO) was necessary for the complete reduction of acute noxious sensing; single or double KO combinations resulted in deficits in heat responsiveness, but mice still conserved vigorous withdrawal responses to noxious heat [90]. Heat activation of TRPM2 and TRPM5 was obtained in inside-out patches, suggesting a membrane-delimited mechanism. Interestingly, TRPM2 activation seems to result from the increase in the IV slope while that of TRPM4 and TRPM5 results from a shift of the activation curve to negative potentials [87,88]. These last two channels are essentially not permeable to calcium [12,24].

### 3.2. Cold-Sensitive TRP Channels

Two TRP channels are activated by decreases in temperature (Figure 1), TRPM8 (<25 °C) activates in the cool range while TRPA1 (<18 °C) senses cold-painful temperatures [23,25,91,92]. Similarly, cool fibers (Aδ and C) have activation thresholds at about 30 °C and cold fibers (C) have activation thresholds <20 °C. Accordingly, two populations of TG neurons were described in terms of their activation threshold when temperatures decrease: 30 and 20 °C for a low and high threshold, respectively [93]. In general, cold fibers fire continuously at normal skin temperatures and they increase their firing frequency when the skin is cooled down, or they shut down when the skin is warmed. In addition, cold fibers can adapt to small decreases in temperature [25]. 

**Figure 3:**
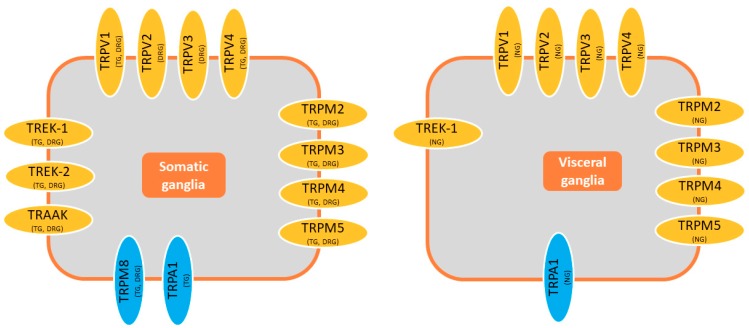
Most temperature-modulated channels are expressed in both somatic and visceral afferents. The expression of TREK2, TRAAK, and TRPM8 in visceral ganglia seems to be low when compared with other channels; however, colocalization of all these channels in the same neuron should be investigated in order to have a real picture of their relative importance in temperature sensing.

TRPM8 channels are voltage-dependent cationic channels that are permeable to Na^+^, K^+^, Cs^-^_,_ and Ca^2+^ (P_Ca_/P_Na_ ~3) [24,92,94]. When expressed in HEK cells and recorded in whole-cell configuration, they show a voltage-dependent outwardly rectifying current that strongly increases upon cooling from 30 to 15 °C or through the application of menthol. Importantly, both basal and cold-stimulated currents reverse around 0 mV and were almost negligible below this potential [9,71,92]. Cooling CHO cells expressing TRPM8 (in the range of 25 to 15 °C) also induces an increase in intracellular calcium [92], and the Q10 in the range of 25 to 18 °C is around 24. The effect of temperature is due to an increase in the open probability and a shift in the conductance–voltage relationships along the voltage axis [9]. Similar results were obtained in inside-out macropatch recordings, although the stimulation occurred at lower temperatures, suggesting that the integrity of the cell is important but not indispensable [71]. The role of this channel as a detector of painful cold has been questioned in experiments on KO mice, but nevertheless, it is accepted that it is an important cold sensor in vagal, TG, and especially DRG afferents [95,96,97,98]. It was predicted that cold transduction may require the activation and inhibition of several different ion channels (see Figure 3), such as TRP, TREK, and ENaC channels [93]. If this were the case, TRP channels would probably be more important in the noxious-cold range, whereas TREK channels might participate more strongly in the cool range of temperatures (Figure 1). TRPM8 is expressed in small-diameter DRG and TG neurons, presumably thermoreceptors, yet it seems not to co-localize with TRPV1 [25,92,94].

TRPA1 is activated by lower temperatures than TRPM8 (<18 °C), and while it would be expected to be involved in cold nociception, this is not that clear [91,98,99,100,101]. TRPA1 generates an outward rectifying cationic current, both in control conditions and when cold activated (about 10 °C), with similar permeability for Ca^2+^ and Na^+^ (P_Ca_/P_Na_ ~1) [24,76,91]. Cinnamaldehyde can selectively activate currents through this channel in native DRG neurons, as can bradykinin (when co-expressed with BK receptors), strongly suggesting a role in sensing nociceptive stimuli [91]. However, TRPA1 KO mice do not seem to have difficulties in sensing cold stimuli through the skin, while the response of TRPM KO mice to cold is significantly dampened [96,97,101]. By contrast, about 50% of NG neurons in culture were activated by cooling (<24 °C), mainly through TRPA1 channel activation (increase in [Ca]_i_, depolarization, and AP firing). Interestingly, about 10% of the NG neurons responded to cold through a TRPA1- and Ca-independent pathway [95]. TRPA1 often co-localizes with TRPV1, and in fact, this could explain the paradoxical hot sensation experienced with an extremely cold stimulus [23,100]. Interestingly, most NG neurons sensitive to cold are also sensitive to heat [95]. TRPA1 is expressed in DRG and TGs, while TRPA1 and TRPM8 are not co-expressed in DRG neurons [91,99], but they are in TG neurons [90]. In summary, the data available suggest that TRPA1 is the principal ion channel involved in cold sensation in visceral (NG) neurons, while TRPM8 would fulfil the same role in somatic neurons [95].

### 3.3. Molecular Origin of Thermosensitivity

The mechanism by which temperature modulates TRP channels is still unclear, yet several hypotheses have been proposed: (1) changes in temperature could produce a ligand that binds to a receptor and affects the channel; (2) changes in temperature could produce a structural change in the channel that provokes its opening; (3) temperature changes could affect the structure of the membrane, causing changes in tension that would in turn affect ion channels. Because capsaicin induces burning pain, it has been hypothesized that both capsaicin and heat may use a common mechanism to activate TRPV1 and produce pain. Both stimuli affect excised patches, and in general, it is accepted that TRPV1 is directly activated by noxious-heat, so that it can be considered a true heat sensor [23].

The fact that TRPM8 can be activated by cooling in inside-out patches suggests that the mechanism is membrane delimited, also arguing against the participation of a second messenger pathway [71]. Notwithstanding, inhibition of phospholipase C strongly dampened the increase in calcium provoked by cold stimuli in TRPM8-expressing CHO cells [91]. Cooling activates TRPM8-expressed channels by causing a shift in the voltage dependence of activation to negative values, and the same mechanism is responsible for the activation of TRPV1 by heat [9,11,71] but not the activation of TRPM2 [88]. It has been proposed that temperature induces large rearrangements of the protein and thus, the existence of a temperature-sensing domain or “temperature sensor” in the structure of TRPM8 channels [9]. Much like for TRPM8, inhibition of phospholipase C strongly reduces the increase in calcium provoked by cold stimuli in TRPA1-expressing CHO cells [90].

## 4. Conclusions

There is already sufficient data supporting the role of TREK and TRP channels in two fundamental aspects of the response to temperature. On the one hand, they are responsible for capturing the thermal sensation at the peripheral level, acting as thermosensors in thermoreceptive neurons or keratinocytes. On the other hand, yet no less important, they help regulate body temperature and allow neurons in the hypothalamus to become internal thermoreceptors. There are several important differences between the two main types of thermosensor channels that have been reviewed in this article (see Figure 2). First, TREKs are potassium channels with negative reversal potentials, such that their activation would result in a reduction in thermoreceptor activity. By contrast, as the reversal potential of TRPs (cationic channels) is close to 0 mV, their activation will result in increased thermoreceptor excitability. Second, the three TREK channels appear to increase their open probability as temperatures increase, while there are two possible situations in the case of TRPs: one in which its activity increases by increasing the temperature; and another in which activity increases when the temperature decreases (Figure 2). Although the activation of these channels generates opposing effects on thermoreceptors (depolarization versus hyperpolarization), the information available to date regarding the participation of TRP and TREK channels in thermosensitivity strongly suggests that both types of channels collaborate and complement each other to generate the sensations of heat, cold, and thermal pain. In support of this hypothesis, TREK and TRP channels are very often co-expressed in thermoreceptors and other sensory neurons [55,102]. TRP channels are generally accepted as the primary thermosensors; however, several lines of evidence indicate that other channels are necessary to explain the full plethora of mechanisms involved in thermosensation. TREK2 channels appear to be important in thermoreception at moderate temperatures and sensing innocuous cold but not aversive cold, while TREK1 and TRAAK acting together may be important in sensing painful cold.

## Abbreviations

COS	Cell line derived from kidney
DRG	Dorsal root ganglion
HEK	Human embryonic kidney
K2P	Two-pore domain potassium channels
NG	Nodose ganglion
NS	Nervous system
PKA	Protein kinase A
TG	Trigeminal ganglion
TRAAK	TWIK-related arachidonic acid-activated potassium channel
TREK	TWIK-related potassium channel
RMP	Resting membrane potential
